# AI-assisted image analysis of tumor-infiltrating lymphocytes as a prognostic marker in chemotherapy-naïve luminal breast cancer

**DOI:** 10.1038/s41523-026-00999-w

**Published:** 2026-07-01

**Authors:** Dusan Rasic, Sandra Sinius Pouplier, Maj-Britt Jensen, Bent Ejlertsen, Roberto Salgado, Pekka Ruusuvuori, Johan Hartman, Mattias Rantalainen, Anne-Vibeke Lænkholm

**Affiliations:** 1 Department of Surgical Pathology, Zealand University Hospital, Koge, Denmark; 2 Department of Clinical Medicine, Faculty of Health and Medical Sciences, University of Copenhagen, Copenhagen, Denmark; 3 Danish Breast Cancer Group, Department of Oncology, Rigshospitalet, Copenhagen University Hospital, Copenhagen, Denmark; 4 Department of Oncology, Centre for Cancer and Organ Disease, Rigshospitalet, Copenhagen University Hospital, Copenhagen, Denmark; 5 Department of Pathology, ZAS Hospitals, Antwerp, Belgium; 6 Division of Research, Peter MacCallum Cancer Centre, Melbourne, Australia; 7 Institute of Biomedicine, University of Turku, Turku, Finland; 8 Department of Oncology and Pathology, Karolinska Institute, Stockholm, Sweden; 9 Department of Clinical Pathology and Cancer Diagnostics, Karolinska University Hospital, Stockholm, Sweden; 10 Department of Medical Epidemiology and Biostatistics, Karolinska Institute, Stockholm, Sweden; 11 Department of Pathology, Herlev and Gentofte University Hospital, Herlev, Denmark

**Keywords:** Biomarkers, Cancer, Computational biology and bioinformatics, Oncology

## Abstract

In triple-negative and HER2-positive breast cancer, tumor-infiltrating lymphocytes (TILs) are established predictive and prognostic biomarkers, but their role in luminal subtypes remains unclear. We investigated the prognostic significance of TILs using AI-based image analysis in 2298 luminal breast cancers from the DBCG99c cohort, classified by PAM50. Stromal (sTIL) and intraepithelial (iTIL) densities were quantified automatically using a commercial platform. Higher stromal TIL infiltration was associated with improved overall survival, particularly within the first 5 years (heterogeneity *p* = 0.01). In multivariable models, higher sTILs independently predicted lower risk of distant or any recurrence (sHR = 0.95, 95% CI 0.91–0.99) and improved survival (HR = 0.91, 95% CI 0.85–0.97 early; HR = 0.99, 95% CI 0.97–1.00 late). Intraepithelial TILs were not prognostic. No significant interactions were observed by molecular subtype, nodal status, or grade, although a nonsignificant trend toward stronger iTIL effects appeared in luminal B tumors. AI-based TIL quantification thus provides independent prognostic information in high-risk luminal breast cancer.

## Introduction

Tumor-infiltrating lymphocytes (TILs) reflect the host immune response to cancer and have long been studied as a prognostic marker in breast cancer (BC). In patients with early triple-negative breast cancer (TNBC), a high TILs level is significantly associated with improved survival (level 1b evidence)^1^.

Similarly, several studies show that a high baseline level of TILs correlates with pathological complete response (pCR) rates after neoadjuvant chemotherapy in both TNBC and HER2-positive BC^2–6^. Estrogen receptor (ER)-positive, HER2-normal (“luminal”) BC, in contrast, has more immune-cold microenvironment with fewer immune cells and active immunosuppression, and evidence for TILs is still sparse and contradictory^7–10^. Criscitiello et al. observed that higher TILs had a beneficial effect on disease-free survival in younger women, grade 3 BCs, and patients treated with chemotherapy compared to chemotherapy-naïve^11^. Other studies examining broader luminal cohorts have found no clear or consistent association between TILs levels and prognosis^2,3,12,13^. In the meta-analysis by Gao et al., higher levels of TILs were linked to worse outcomes, such as poorer overall survival (OS)^14,15^. These contradictions suggest that, in luminal disease, immune infiltration may sometimes mark a biologically aggressive or treatment-refractory phenotype rather than an effective anti-tumor response.

A critical yet often overlooked driver of TILs behavior in luminal BC is the immune response to endocrine therapy. Estrogen supports an immunosuppressive environment by suppressing dendritic-cell priming, expanding regulatory T cells (Tregs) and myeloid-derived suppressor cells (MDSCs), and blunting CD8⁺ and NK-cell cytotoxicity^16^. Tamoxifen and other selective estrogen degraders (SERDs) exert a double-edged influence: they may upregulate TGF-β, transform CD4⁺ cells toward Tregs/TH2 and induce tumor PD-L1, while at the same time improving NK-cell antibody-dependent cytotoxicity and dendritic recruitment^16–18^. Sustained estrogen blockade with aromatase inhibitors more consistently lowers Treg density, attracts CD8⁺ T cells, and pushes cytokines toward a TH1 profile, potentially converting “cold” tumors into more “immune-hot” lesions^16,19^.

These findings illustrate that the prognostic significance of TILs in luminal BC is not straightforward. Instead of acting as a consistently favorable marker, TILs may signal different biological or clinical implications depending on treatment context and other tumor characteristics. This underscores the importance of further stratified analyses and studies to clarify when, and in which patients with luminal BC, TILs can be considered a meaningful prognostic biomarker.

The evaluation of TILs has been standardized by the TILs Working Group (WG)^20^. The assessment reflects the average TILs density across the entire tumor area, rather than focusing on hotspots. Consensus guidelines prioritize stromal TILs (sTILs) because intraepithelial lymphocytes (iTILs) are fewer, more heterogeneous, and difficult to score reproducibly on routine HE-slides^20^; however, even though their counts usually parallel those in the stroma, emerging data show that they might have an independent prognostic value when assessed reproducibly^21,22^.

However, manual TILs scoring is a time-consuming task and relies heavily on the judgment of the individual pathologist^20^. There is a need to evaluate whether AI-based tools may help address some of the practical limitations associated with manual scoring. Digital image analysis (DIA) tools^22,23^ offer a potential approach to quantifying TILs in BC.

In this study, we aimed to evaluate the prognostic value of stromal and intraepithelial TILs assessed by DIA in patients with luminal BC. Additionally, we investigated the association between TILs and clinical outcomes within the luminal A and luminal B subgroups, as well as nodal status and grade interaction.

## Results

**Fig. 1:**
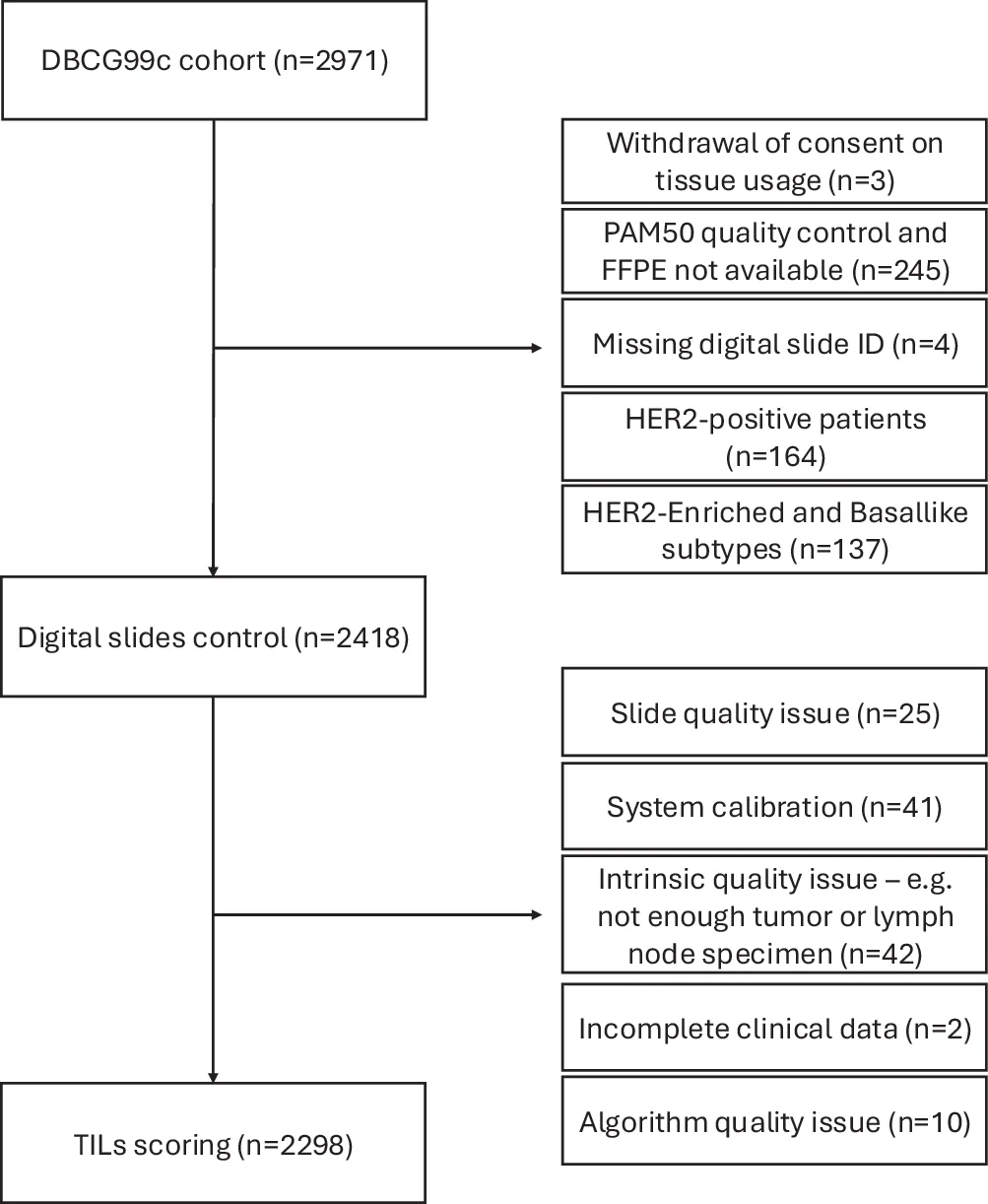
Flow diagram. Flow diagram of sample selection for TILs assessment in the DBCG99c cohort.

Figure 1 shows the study flowchart. 2298 slides from the same number of patients were included for the final TILs analysis. Median sTIL and iTIL densities on the whole population level were 1.66 (IQR 0.97–3.06) and 0.22 (IQR 0.10–0.49) cells per 0.01 mm^2^, respectively. The scatterplot comparing digitally quantified sTIL density with pathologist-assessed percentages reveals a fair alignment between the two scoring methods, reflecting good concordance with a Spearman rank correlation of ρ = 0.52 (*p* < 0.01) (Supplementary Fig. 1). Comparison of scaled digitally quantified sTIL density with pathologist-assessed sTIL percentages showed an intraclass correlation coefficient of 0.74 (95% CI 0.64–0.81). The distributions of AI-derived and pathologist-assessed sTIL scores are shown in Supplementary Fig. 2.

### Association of TILs variables with clinicopathological factors

Both high sTILs and iTILs were associated with a younger age at diagnosis (*p* = 0.01) (Table 1). In luminal A tumors, a higher proportion of low sTILs cases was seen, compared with luminal B tumors (*p* < 0.01). Infiltration by sTILs was also significantly associated with histological grade, with grade 3 showing the highest proportion of high sTILs cases (63.5% in grade 3 compared to 52.5 and 39.4% for grades 2 and 1, respectively, *p* < 0.01). iTILs did not vary significantly with the Nottingham grade (*p* = 0.13). Lobular subtypes often had lower sTILs, but conversely higher iTILs (*p* < 0.01). Presence of vascular invasion was also significantly associated with high sTILs, but not iTILs (*p* < 0.01, and *p* = 0.42, respectively). Other clinicopathological factors (ER, lymph node status, and tumor size) did not show significant associations with neither sTILs nor iTILs densities.

**Table 1:** Association of sTILs and iTILs with clinicopathological factors

Variable	Level	Low sTILs (*n* = 1149)	High sTILs (*n* = 1149)	*p*-value	Low iTILs (*n* = 1149)	High iTILs (*n* = 1149)	*p* value
Age (years)	50–59	397 (47.5)	438 (52.5)	0.01	393 (47.1)	442 (52.9)	0.01
	60–69	481 (49.3)	495 (50.7)		485 (49.7)	491 (50.3)	
	≥70	271 (55.6)	216 (44.4)		271 (55.6)	216 (44.4)	
Vascular invasion	No	1046 (51.0)	1003 (49.0)	<0.01	1031 (50.3)	1018 (49.7)	0.42
	Yes	103 (41.4)	146 (58.6)		118 (47.4)	131 (52.6)	
Positive lymph nodes	0	510 (49.3)	524 (50.7)	0.88	519 (50.2)	515 (49.8)	0.66
	1	359 (51.0)	345 (49.0)		340 (48.3)	364 (51.7)	
	2	183 (50.7)	178 (49.3)		188 (52.1)	173 (47.9)	
	3	97 (48.7)	102 (51.3)		102 (51.3)	97 (48.7)	
Tumor size (mm)	≤10	107 (50.2)	106 (49.8)	0.06	107 (50.2)	106 (49.8)	0.75
	11-20	464 (47.3)	517 (52.7)		502 (51.2)	479 (48.8)	
	21-30	396 (51.0)	381 (49.0)		383 (49.3)	394 (50.7)	
	>30	182 (55.7)	145 (44.3)		157 (48.0)	170 (52.0)	
Molecular subtype	Luminal A	744 (53.5)	646 (46.5)	<0.01	663 (47.7)	727 (52.3)	<0.01
	Luminal B	405 (44.6)	503 (55.4)		486 (53.5)	422 (46.5)	
NHG	1	393 (60.6)	256 (39.4)	<0.01	330 (50.8)	319 (49.2)	0.13
	2	665 (47.5)	735 (52.5)		681 (48.6)	719 (51.4)	
	3	91 (36.5)	158 (63.5)		138 (55.4)	111 (44.6)	
Estrogen receptor	10–59	103 (49.8)	104 (50.2)	0.25	93 (44.9)	114 (55.1)	0.15
	60–89	240 (51.4)	227 (48.6)		245 (52.5)	222 (47.5)	
	90–99	283 (46.8)	322 (53.2)		289 (47.8)	316 (52.2)	
	100	513 (51.7)	480 (48.3)		512 (51.6)	481 (48.4)	
	Positive*	10 (38.5)	16 (61.5)		10 (38.5)	16 (61.5)	
Histological subtype	Ductal	922 (48.3)	986 (51.7)	<0.01	1,015 (53.2)	893 (46.8)	<0.01
	Lobular	177 (56.2)	138 (43.8)		89 (28.3)	226 (71.7)	
	Other	50 (66.7)	25 (33.3)		45 (60.0)	30 (40.0)	
DR follow-up time	Years (IQR)	9.2 (7.0; 10.1)	9.2 (7.2; 10.1)		9.1 (6.5;10.1)	9.6 (7.5;10.1)	
DR events (%)	No	865 (49.8)	872 (50.2)		866 (49.9)	871 (50.1)	
	Competing	173 (53.4)	151 (46.6)		173 (53.4)	151 (46.6)	
	Yes	111 (46.8)	126 (53.2)		110 (46.4)	127 (53.6)	
TR follow-up time	Years (IQR)	9.2	9.2		9.1	9.5	
		(7.0; 10.1)	(7.2; 10.1)		(6.5; 10.1)	(7.5; 10.1)	
TR events (%)	No	852 (49.7)	864 (50.3)		854 (49.8)	862 (50.2)	
	Competing	170 (53.1)	150 (46.9)		170 (53.1)	150 (46.9)	
	Yes	127 (48.5)	135 (51.5)		125 (47.7)	137 (52.3)	
OS follow-up time	Years (IQR)	21.9 (21.0; 22.9)	21.8 (21.0; 22.9)		21.7 (21.0; 22.9)	21.9 (21.0; 22.9)	
OS events (%)	No	429 (46.2)	499 (53.8)		455 (49.0)	473 (51.0)	
	Yes	720 (52.6)	650 (47.4)		694 (50.7)	676 (49.3)	

Values in the parentheses are given as row percentages.

*sTILs* stromal tumor-infiltrating lymphocytes, *iTILs* intraepithelial tumor-infiltrating lymphocytes, *NHG* Nottingham histological grade, *DR* distant recurrence, *TR* any recurrence, *OS* overall survival, *IQR* interquartile range.

*Value on continuous scale unknown.

Luminal B tumors were associated with worse prognostic factors and were more often larger, higher grade, and showed vascular invasion (all *p* < 0.05) (Supplementary Table 1), while luminal A tumors were more commonly lobular (*p* < 0.01), which may partly reflect the study inclusion criteria (Supplementary Table 1).

### Survival analysis

**Fig. 2:**
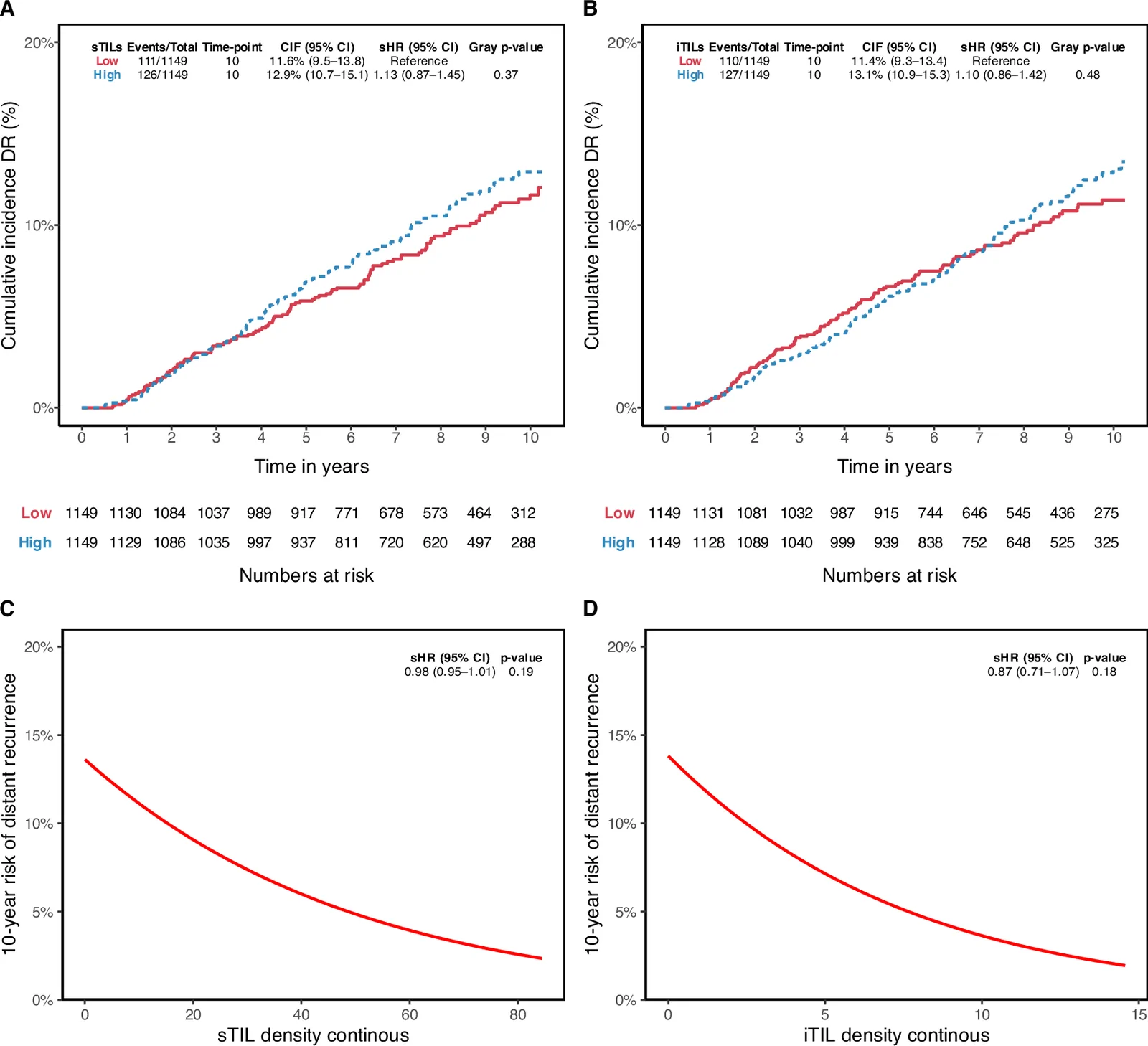
Distant recurrence by sTIL and iTIL categories and levels. Cumulative incidence for DR by sTIL category (**A**), and iTIL category (**B**). 10-year risk of distant recurrence as a function of sTIL (**C**) and iTIL (**D**). sHR subdistribution hazard ratio from univariable Fine–Gray model.

**Fig. 3:**
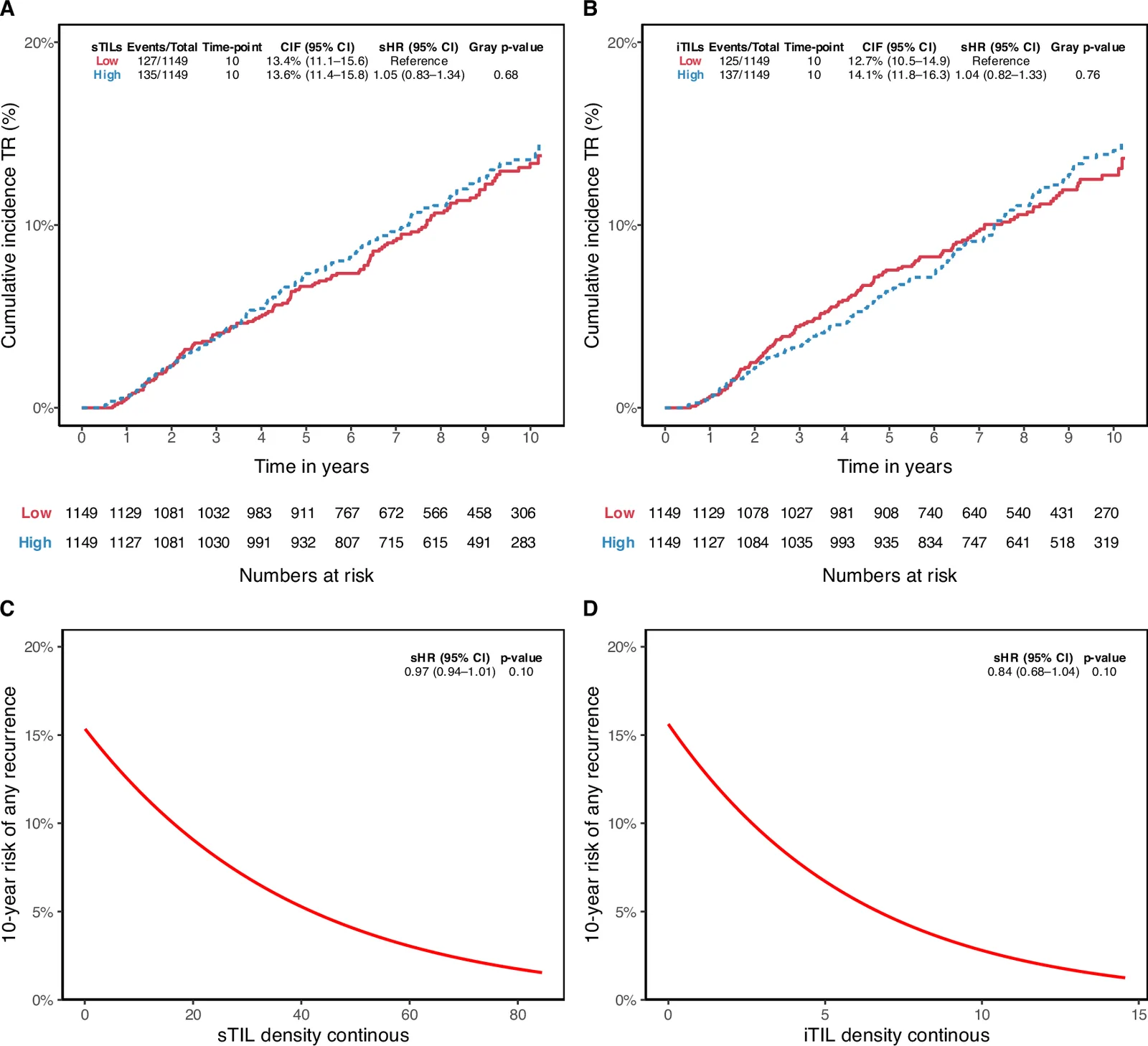
Any recurrence by sTIL and iTIL categories and levels. Cumulative incidence for TR by sTIL category (**A**), and iTIL category (**B**). 10-year risk of any recurrence as a function of sTIL (**C**) and iTIL (**D**). sHR subdistribution hazard ratio from Fine–Gray model.

**Fig. 4:**
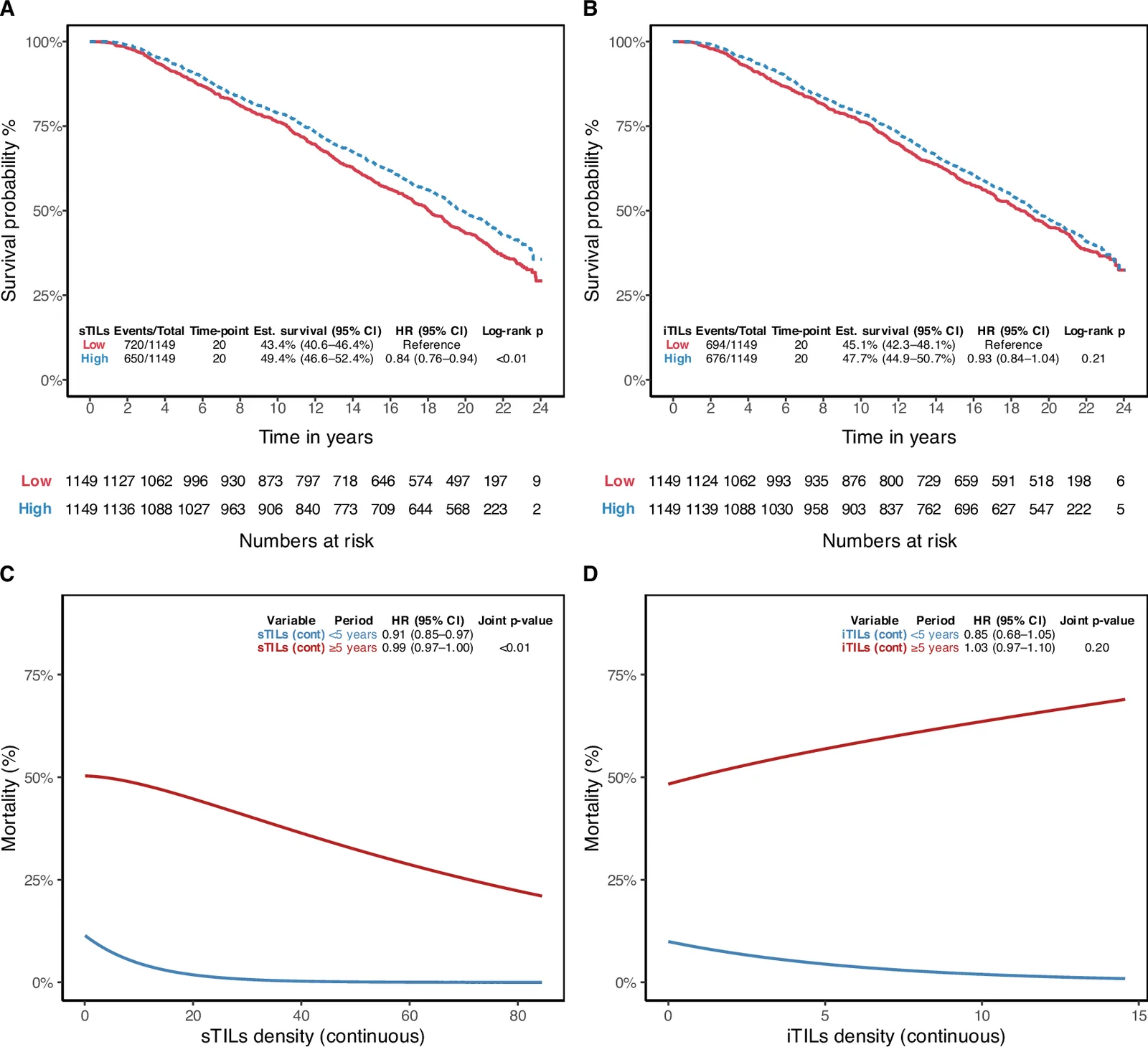
Overall survival by sTIL and iTIL categories and levels. Association of TIL density metrics with overall survival. Kaplan–Meier estimates of overall survival by sTILs (**A**) and iTILs (**B**). Predicted cumulative mortality by sTIL (**C**) and iTIL (**D**) density from a univariable piecewise-Cox model allowing the hazard slope to differ before and after 5 years. The solid blue line shows the probability of death by 5 years (0–5 y cumulative), and the red line shows the probability of death between years 5–20 conditional on surviving to year 5 (≥5 yr conditional). OS overall survival, HR hazard ratio, *p,* *p* value from log-rank (**A**, **B**) and Wald tests with 2 degrees of freedom testing whether both coefficients are 0 simultaneously (**C**, **D**).

In univariable analysis, no significant associations were found for time to distant recurrence (DR) or time to any recurrence (TR) (Figs. 2A, C and 3A, C). Similarly, no statistically significant association between iTILs and any analysed endpoints was observed (Table 2 and Figs. 2–4). For overall survival (OS), higher levels of sTILs were associated with longer survival (*p* < 0.01), both when modeled on a continuous scale and as a categorical variable (Table 2 and Fig. 4). The effect was predominant in the first 5 years after diagnosis (test of heterogeneity *p* = 0.02).

**Table 2:** Associations between distant recurrence, any recurrence, and overall survival with sTILs and iTILs (continuous and categorical): univariable and multivariable analyses

Univariable analysis							
	DR		TR		OS		
Variable	sHR (95% CI)	*p* value	sHR (95% CI)	*p* value	HR (95% CI)		*p* value
sTILs (cont.)	0.98 [0.95;1.01]	0.19	0.97 [0.94;1.01]	0.10	<5 y	0.91 [0.85;0.97]	*<0.01
					≥5 y	0.99 [0.97;1.00]	
sTILs (High vs Low)	1.13 [0.87;1.45]	0.37	1.05 [0.83;1.34]	0.68	0.84 [0.76;0.94]		<0.01
iTILs (cont.)	0.87 [0.71;1.07]	0.18	0.84 [0.68;1.04]	0.10	<5 y	0.85 [0.68;1.05]	*0.20
					≥5 y	1.03 [0.97;1.10]	
iTILs (High vs Low)^a^	1.10 [0.86;1.42]	0.48	1.04 [0.82;1.33]	0.76	0.93 [0.84;1.04]		0.21
**Multivariable analysis**							
	DR		TR		OS		
sTILs (cont.)	0.95 [0.91;0.99]	0.03	0.95 [0.91;0.99]	0.01	<5 y	0.91 [0.85;0.97]	*<0.01
					≥5 y	0.99 [0.97;1.00]	
sTILs (High vs Low)	0.98 [0.75;1.27]	0.87	0.93 [0.73;1.20]	0.59	0.87 [0.78;0.97]		0.01
iTILs (cont.)	0.82 [0.65;1.04]	0.11	0.80 [0.64;1.02]	0.07	<5 y	0.85 [0.69;1.04]	*0.22
					≥5 y	1.02 [0.96;1.09]	
iTILs (High vs Low)^a^	1.08 [0.83;1.42]	0.56	1.04 [0.81;1.34]	0.76	0.96 [0.86;1.07]		0.50

Recurrence is analysed with Fine–Gray subdistributional hazard model and results are reported with subdistribution hazard ratios (sHR). Overall survival is analysed with a Cox proportional hazard model—results are hazard ratios (HR).

*DR* distant recurrence, *TR* any recurrence, *OS* overall survival, *sHR* subdistribution hazard ratio, *HR* hazard ratio.

^a^DR model for iTIL (categorical) marginally violated the PH assumption, but for the sake of consistency, kept as is.

*p values from the Wald test with 2 degrees of freedom (whether there is an effect of sTILs and iTILs on the outcome overall).

In the multivariable analysis (Table 2), continuous sTIL density was significantly associated with all endpoints (*p* = 0.03 for DR, 0.01 for TR, and <0.01 for OS). Similarly to univariable analysis, the benefit of higher sTILs on OS was stronger in the first 5 years. Categorical sTILs analysis showed that high sTILs were associated with improved OS compared to low sTILs (HR = 0.87, 95% CI: 0.78–0.97, *p* = 0.01), but this observation did not hold for DR or TR. Intraepithelial TIL density was not significantly associated with any endpoint, although a nonsignificant tendency toward improved DR (*p* = 0.11) and TR (*p* = 0.07) was observed.

### Subtype interaction model

We then examined whether the prognostic effect of TILs differed by intrinsic subtype, as defined by the Prosigna PAM50 assay, rather than by immunohistochemistry (IHC) surrogates. For both DR and TR, the estimated subdistribution hazard ratios (sHR) per unit increase in sTILs were nearly identical across luminal A and luminal B tumors, and the formal test for interaction confirmed non-heterogeneity (*p* = 0.92 and *p* = 0.99, respectively), indicating no evidence that the sTIL effect varied by subtype (Table 3). Likewise, in a Cox model for OS, no evidence of a differential effect between luminal A and luminal B tumors (*p* = 0.85) was observed, including when considering the early (<5 years) and late (≥5 years) time periods (Table 3).

**Table 3:** Associations between distant recurrence, any recurrence, and overall survival with sTILs and iTILs (continuous): multivariable analyses with interaction by subtype (Luminal A vs Luminal B)

Subgroup analysis – subtype								
		DR		TR		OS		
Variable	subtype	sHR (95% CI)	*p*_heterogeneity_	sHR (95% CI)	*p*_heterogeneity_	HR (95% CI)		*p*_heterogeneity_
sTILs (cont.)	LumA	0.96 [0.88;1.04]	0.92	0.95 [0.88;1.03]	0.99	<5 y	0.91 [0.81;1.02]	0.85
						≥5 y	0.98 [0.95;1.01]	
	LumB	0.95 [0.91;1.00]		0.95 [0.90;1.00]		<5 y	0.91 [0.84;0.98]	
						≥5 y	0.99 [0.97;1.01]	
iTILs (cont.)	LumA	1.00 [0.73;1.36]	0.08	0.96 [0.71;1.29]	0.09	<5 y	1.00 [0.80;1.26]	0.18
						≥5 y	1.02 [0.93;1.11]	
	LumB	0.69 [0.52;0.91]		0.67 [0.50;0.89]		<5 y	0.65 [0.43;0.98]	
						≥5 y	1.03 [0.94;1.12]	

Recurrence is analysed with Fine–Gray subdistributional hazard model and results are reported with subdistribution hazard ratios (sHR). Overall survival is analysed with a Cox proportional hazards model—results are hazard ratios (HR).

*p*_hetereogeneity_ indicates Wald test with 1 degree of freedom – prognostic value of given variable is different between subgroups if *p* < 0.05, and for OS variables Wald test with 2 degrees of freedom (tests whether the hazard ratios for sTILs/iTILs differ by subtype in either time period (i.e., HR (<5 y, Luminal A) = HR (<5 y, Luminal B) and HR (≥5 y, Luminal A) = HR (≥5, Luminal B)).

*DR* distant recurrence, *TR* any recurrence, *OS* overall survival, *sHR* subdistribution hazard ratio, *HR* hazard ratio.

Subdistribution HRs for intraepithelial TILs appeared lower in luminal B compared to luminal A tumors, and the formal test for interaction showed borderline evidence of heterogeneity between subtypes (*p* = 0.08 and *p* = 0.09 for DR and TR, respectively). For OS, numerical estimates suggested that higher iTILs were associated with lower hazards in luminal B tumors during the first 5 years of follow-up; however, the overall Wald test for heterogeneity was not significant (*p* = 0.18) (Table 3).

### Nodal status interaction model

For the DR endpoint, continuous sTIL density showed no clear effect in node-negative patients (sHR 0.98, 95% CI 0.94–1.02) but was modestly protective in node-positive cases (sHR 0.93, 95% CI 0.86–0.99) (Table 4). A similar pattern emerged for the TR endpoint, with no association in node-negative tumors (sHR 0.97, 95% CI 0.93–1.01) and a borderline benefit in node-positive tumors (sHR 0.93, 95% CI 0.87–0.99) (Table 4). However, no statistically significant difference between node-negative and node-positive patients was observed (heterogeneity *p* = 0.15, and 0.19 for DR and TR, respectively). For OS, higher sTIL density was modestly protective during the first 5 years in both node‑negative and node‑positive patients, but showed no impact after 5 years in either group, and there was no evidence that this early benefit differed by nodal status (*p* = 0.62) (Table 4).

**Table 4:** Associations between distant recurrence, any recurrence, and overall survival with sTILs and iTILs (continuous): multivariable analyses with interaction by nodal status (node-negative and node-positive disease)

Subgroup analysis – Nodal status								
		DR		TR		OS		
Variable	Nodal status	sHR (95% CI)	*p*_heterogeneity_	sHR (95% CI)	*p*_heterogeneity_	HR (95% CI)		*p*_heterogeneity_
sTILs (cont.)	Neg	0.98 [0.94;1.02]	0.15	0.97 [0.93;1.01]	0.19	<5 y	0.89 [0.81;0.97]	0.62
						≥5 y	0.99 [0.97;1.01]	
	Pos	0.93 [0.86;0.99]		0.93 [0.87;0.99]		<5 y	0.92 [0.86;0.99]	
						≥5 y	0.99 [0.97;1.01]	
iTILs (cont.)	Neg	0.75 [0.57;0.97]	0.48	0.69 [0.51;0.92]	0.27	<5 y	0.60 [0.38;0.95]	0.10
						≥5 y	0.99 [0.91;1.08]	
	Pos	0.86 [0.63;1.18]		0.86 [0.64;1.15]		<5 y	0.96 [0.78;1.19]	
						≥5 y	1.06 [0.98;1.15]	

Recurrence is analysed with Fine–Gray subdistributional hazard model and results are reported with subdistribution hazard ratios (sHR). Overall survival is analysed with a Cox proportional hazards model—results are hazard ratios (HR).

*p*_hetereogeneity_ indicates Wald test with 1 degree of freedom – prognostic value of given variable is different between subgroups if *p* < 0.05, and for OS continuous variables Wald test with 2 degrees of freedom (tests whether the hazard ratios for sTILs/iTILs differ by nodal status in either time period (i.e. HR (<5 y, node negative) = HR (<5 y, node positive) and HR (≥5 y, node negative) = HR (≥5, node positive)).

*DR* distant recurrence, *TR* any recurrence, *OS* overall survival, *sHR* subdistribution hazard ratio, *HR* hazard ratio.

Intraepithelial TIL density was associated with an sHR of 0.75 (95% CI 0.57–0.97) for DR in node-negative patients and 0.86 (95% CI 0.63–1.18) in node-positive patients. For TR, the corresponding sHRs were 0.69 (95% CI 0.51–0.92) and 0.86 (95% CI 0.64–1.15). There was no evidence that the effect differed by nodal status, neither for DR nor TR endpoints. For OS, node-negative patients showed HR of 0.60 (95% CI 0.38–0.95) in the first 5 years and HR of 0.99 (95% CI 0.91–1.08) thereafter. In node-positive patients, the corresponding HRs were 0.96 (95% CI 0.78–1.19) in the first 5 years and 1.06 (95% CI 0.98–1.15) after 5 years. However, the formal test for heterogeneity by nodal status with respect to OS was not statistically significant (*p* = 0.10).

### Grade interaction model

In an exploratory analysis, we examined whether the prognostic effect of TILs differed by histological grade. No significant grade interaction was observed for sTILs or iTILs across any endpoint (Supplementary Table 2). Point estimates for sTILs were uniform across grade groups for DR and TR, and the early-period OS benefit of sTILs was present across all three grade groups, with only modest differences in magnitude. For iTILs, a trend toward stronger benefit with increasing grade was observed, but formal tests did not support grade heterogeneity.

## Discussion

In this study, we analyzed stromal and intraepithelial TILs in a cohort of 2298 luminal BC patients using commercial DIA tools that automatically define regions of interest and quantify immune cell counts, replicating the TIL-WG recommended workflow^20^.

Our results suggest better outcomes for luminal cancers with higher sTIL levels, especially for OS in the first 5 years of follow-up. For distal and any recurrence endpoints, this only reached statistical significance after adjusting for other clinicopathological factors, suggesting a complex interplay between different biologies. Although the point estimates suggested a tendency toward greater benefit in luminal B disease, our study cannot provide statistically robust evidence of subtype-specific effects (nonsignificant heterogeneity tests). Generally, the finding of a favorable independent prognostic effect of sTILs in this luminal cohort may appear at odds with studies reporting neutral or adverse associations^2,12,21^. This apparent contradiction may be explained by confounding, as in luminal breast cancer, sTILs are enriched in tumors with aggressive features, e.g., higher histological grade, luminal B subtype, and vascular invasion, which are associated with worse prognosis^7,11,21^. In unadjusted analyses, these features might dominate and obscure the independent effect of TILs. Multivariable adjustment disentangles TILs’ independent contribution from the adverse characteristics of the tumors in which they are enriched, revealing the underlying beneficial association. Similar to our results, a recent study by Cai et al. reported that high AI-assessed TIL abundance is independently associated with improved OS across both luminal A and luminal B subtypes^24^.

It is established that luminal A and luminal B subtypes are distinct entities with substantial differences at the molecular level^25^, and several studies have now highlighted that luminal B tumors exhibit higher TILs infiltration, higher proliferation, and larger genomic instability than luminal A tumors^26,27^. This may reflect a heightened immunogenicity in luminal B cancers; however, the associated immune response does not consistently translate into an improved prognosis. Quite the contrary, luminal B tumors are often associated with worse outcomes, more similar to basal-like and HER2-enriched than luminal A^27^. It is also hypothesized that immune cell infiltration in luminal B tumors may signify an ineffective anti-tumor immune response, potentially driven by immunosuppressive cell populations such as regulatory T cells or by establishing a tumor microenvironment in which cancer cells thrive^26^. In contrast, luminal A tumors are more often characterized by low TILs counts and reduced expression of immune activation markers, aligning with their more favorable clinical outcomes, but in TILs-rich setting, some evidence suggests an association with worse outcomes^12,26,28^. In a larger study, Denkert et al.^2^ showed favorable prognosis in grade 3 ER + /HER2- patients with higher TILs levels and an inverse finding in low-grade tumors. However, those findings came from subgroup-specific models rather than a formal interaction test. Our exploratory grade interaction analysis found no significant heterogeneity across grade groups, and the adjusted sTIL benefit on early OS was consistent across all three grade strata, supporting a grade-independent prognostic value of sTILs in endocrine-treated luminal breast cancer.

Others^11,29^ have also reported that the prognostic effect of TILs may be modulated by proliferative status, as patients with high Ki-67 expression (often indicative of luminal B biology) appear to derive more benefit from a TILs-rich tumor microenvironment. A more recent study by ref. ^30^ showed that the presence of stromal TILs (>0%) was associated with worse relapse-free survival in luminal B BC, with a similar trend for OS. While this differs both from our findings and the recent study by ref. ^24^, an important distinction is that the majority of patients in their cohort received chemotherapy. Treatment modality may influence the prognostic role of TILs, as chemotherapy can substantially reshape the tumor immune microenvironment^31^. In contrast, our exclusively endocrine-treated cohort is not subject to these effects, and in this context, higher sTIL levels may instead reflect a more active and clinically beneficial immune response, consistent with the protective associations observed after multivariable adjustment. Another comparable study by Makhlouf et al. showed that high TILs counts assessed by AI and measured either in stroma or in tumor, were associated with shorter breast cancer-specific survival^21^. However, in their study, counts were not normalized to the area assessed and thus did not fully conform to the recommended TIL-WG guidelines. We could not confirm the negative prognostic value of intraepithelial TILs reported in ref. ^21^, on the contrary, in our cohort, a protective effect in luminal B tumors was observed, similarly to the study by ref. ^24^.

Lastly, we analysed whether distinct patterns according to nodal status were present. Point estimates suggested that higher intraepithelial TIL levels were associated with better outcomes across DR, TR, and short-term OS in node-negative patients, whereas stromal TILs, but not intraepithelial TILs, appeared more protective in node-positive patients. However, none of these differences reached statistical significance in the formal heterogeneity tests. This aligns with a previous study by Loi et al., which failed to demonstrate significant prognostic associations for either stromal or intraepithelial TILs in node-positive disease^32^.

Given that luminal BC constitutes the majority of all BC cases, an important clinical question is whether the TILs levels can be used to stratify risk or inform treatment decisions. Emerging evidence from recent trials underscores the predictive significance of TILs, especially in the context of immunotherapy. For instance, in CheckMate 7FL, a randomized phase 3 trial, patients with higher levels of sTILs showed more benefit from nivolumab compared to immune-cold tumors^33^. Similarly, the phase II GIADA trial specifically targeted luminal B-like BC, characterized by high proliferation (Ki-67 ≥ 20%) and/or high histological grade (grade 3)^34^. That study demonstrated a substantial association between higher TILs densities and increased likelihood of achieving pCR following sequential neoadjuvant anthracycline chemotherapy and nivolumab combined with endocrine therapy. Furthermore, the trial highlighted that luminal B-like tumors exhibiting basal-like molecular characteristics and high immune gene expression signatures were particularly responsive to immunotherapy^34^.

Nevertheless, methodological challenges remain, and even though the TILs Working Group^20^ provided general guidelines for TILs scoring, previous studies in luminal cancer have been highly heterogeneous in methodology and statistical analyses, further complicating the ability to draw consistent conclusions^12^. Moreover, the difficulties encountered in visual assessment of TILs—especially regarding the intraepithelial TILs component—challenge the reproducibility of this method^12,13^. In TILs-rich BC subtypes, this might not translate into a substantial caveat, but in the luminal BC, where immune cells appear in lesser numbers, even minor discrepancies might lead to spurious results. Previous studies have applied DIA on whole-slide images to a variety of materials showing both predictive and prognostic value of TILs assessed by machine^21,22,35–40^. A study by ref. ^41^ even showed that different modeling approaches to digital TILs quantification produce comparable results. Thus, DIA approaches may bridge current methodological gaps toward more reproducible TILs assessment. However, discordant cases, most notably those with pathologist-assessed sTIL of 0% but non-zero DIA-derived density, are still present and likely reflect inherent methodological differences, e.g., lymphocytes present at densities below the visual detection threshold for semi-quantitative scoring may still be captured by automated cell detection, while morphologically ambiguous small round cells such as plasma cells, mast cells, or fibroblasts with condensed nuclei may contribute a small number of false-positive DIA detections.

A limitation of our study is the discrepancy between manually scored TILs percentages by the pathologist and the derived TILs densities generated by the Visiopharm AI application. Within this context, it should be noted that the ICC was computed on min-max normalized values (each variable independently rescaled to [0,1]), meaning it reflects agreement in the normalized domain rather than absolute equivalence in original units, and this estimate is also sensitive to the bounds of the observed range in each measure, since extreme values define the scaling. Cross-study comparability is further complicated by variation in algorithm training, as AI tools developed on different labeled datasets or with different annotation conventions for stromal boundaries and lymphocyte detection will produce systematically offset estimates even on identical slides, adding a further layer of incomparability beyond the unit difference alone^42^. Thus, the present study cannot demonstrate that DIA produces more accurate or objectively truer TIL estimates than manual scoring. The observed concordance is moderate, and the unit mismatch between methods precludes assessment of absolute agreement. However, since the clinical value of TILs ultimately rests on their association with outcome, agreement with manual scoring may not be the only relevant measure of a method’s performance; digital and manual TILs have been reported to retain comparable prognostic value despite imperfect numerical concordance^41^. Additionally, DIA offers reproducibility, and unlike semi-quantitative visual assessment, automated quantification eliminates interobserver variability and can be applied uniformly at scale, which may complement current clinical workflows even if absolute equivalence with manual scoring has not been established. Further limitation of our study is the absence of distinguishing between the immune phenotype of the cells, as TILs are assessed on hematoxylin-eosin (HE) slides. While this is the current standard procedure, it masks underlying biological interactions between different immune cell populations. A study by ref. ^43^ investigated immune phenotypes of TILs by characterizing their FOXP3 and CD8-expression. Adverse outcomes were observed for patients with high FOXP3/CD8 ratios in both ER+ and Luminal A subgroups. Lastly, the ER positivity threshold of ≥10% applied in this cohort reflects the Danish national guidelines at the time of patient enrollment (2000–2003) and is consistent with the published DBCG99c cohort design^44^. The impact of this discrepancy on our results is likely limited, as very few patients would be reclassified at this boundary, but findings should be interpreted in the context of the historical ER definition used.

Our study also has several notable strengths. First, the study has a formal prospective-retrospective design, and our cohort was chemotherapy-naïve, which effectively eliminates the confounding effects of chemotherapy and provides insights specific to endocrine treatment response. Second, access to Danish Breast Cancer Cooperative Group (DBCG)-database enabled us to acquire data with long follow-up period, which is of outmost importance in ER-positive BC thus strengthening the validity of our survival analyses. Finally, instead of relying on surrogate immunohistochemical markers for molecular subtyping, data from the Prosigna PAM50 assay was available for all patients, ensuring a validated (gold standard) and biologically correct classification of subtypes.

To conclude, in this large luminal BC cohort, high stromal TILs were associated with improved overall survival, most notably during the first 5 years after diagnosis. The prognostic importance of TILs appeared context-dependent and influenced by subtype and time since diagnosis. Further research is necessary to clarify the clinical utility of TIL quantification in luminal BC.

## Methods

### Patients

The DBCG99C study population (*N* = 2971) comprises Danish postmenopausal women diagnosed with ER-positive BC during the period from January 2000 to December 2003 who were offered adjuvant endocrine treatment alone. The study has a formal prospective-retrospective design, and the study population was originally selected for investigation of the prognostic impact of the PAM50 for the de-escalation of chemotherapy in lymph node-positive BC^44^. Eligibility required patients to be at least 50 years of age and to fulfill at least one of the following clinical criteria: primary tumor diameter exceeding 20 mm, ductal carcinoma with a histological grade of 2 or 3, or involvement of one to three axillary lymph nodes. Additionally, in the current study, all cases with non-luminal subtypes were excluded (Fig. 1).

ER status was determined by immunohistochemistry with a threshold of 10% for positivity according to national guidelines at the time^45^. HER2 status was determined according to the 2013 ASCO CAP guidelines^46^. Molecular subtype classification was performed using the commercial Prosigna® assay (NanoString Technologies), a diagnostic test based on the PAM50 gene expression signature, applied to formalin-fixed paraffin-embedded tumor tissue as part of the previously published DBCG99c prospective-retrospective study design^44^.

Evaluation for distant metastases involved clinical examination and chest X-ray, with supplementary imaging such as bone scans or skeletal radiographs performed for individuals reporting bone-related symptoms or displaying elevated bone turnover markers. Surgical management consisted of either lumpectomy or mastectomy in combination with sentinel lymph node assessment, followed by axillary dissection in cases of nodal involvement. All included patients were allocated to and received adjuvant endocrine therapy, with a recommended duration of 5 years.

Radiotherapy, delivered as 48 Gy in 2 Gy daily fractions over five days per week, targeted the breast or chest wall in patients under 70 years with tumors larger than 50 mm, and included regional lymph nodes in those with nodal metastases. Detailed descriptions of the DBCG99 cohort protocols have been previously published^44,47^.

### Slide scanning and quality control of digital slides

For each patient, two HE-stained slides of the surgical specimens were selected for analyses. A manual quality control process was conducted, during which slides were reviewed and excluded if the staining quality or the presence of artifacts deemed them inadequate. A single slide per patient was chosen for digitization (40X) using WSI-scanners NanoZoomer S360 and Nanozoomer XR (Hamamatsu Photonics K.K., Shizuoka, Japan).

### Digital image analysis of TILs

**Fig. 5:**
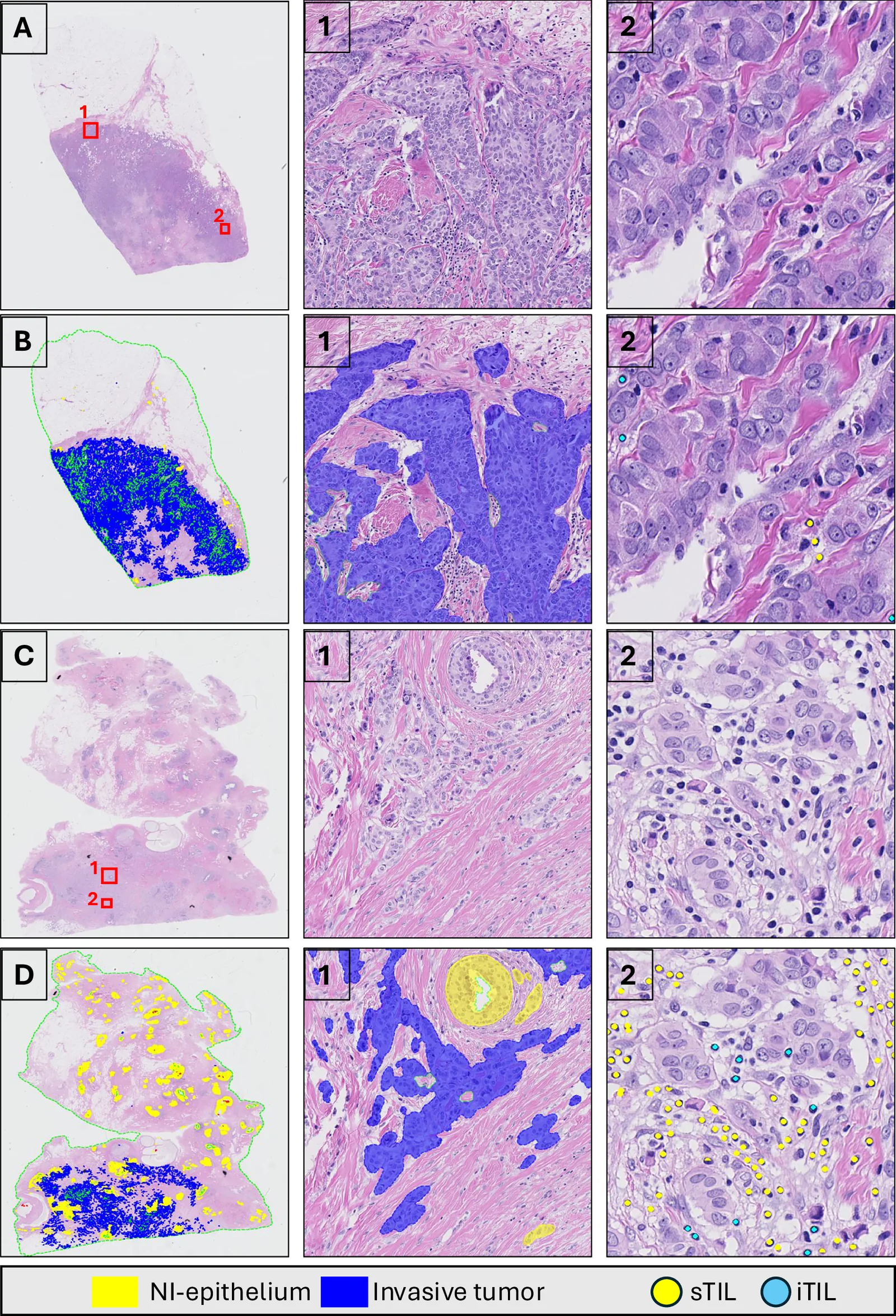
Overview of Visiopharm’s application for TILs assessment in BC WSIs. Panels (**A**, **C**) show original H&E-stained tissue sections with indicated regions of interest (red boxes). Panels (**B**, **D**) demonstrate the corresponding automatic tissue segmentation results, distinguishing invasive tumor regions (blue) from non-invasive epithelium (NI-epithelium, yellow). Magnified views in columns 1 and 2 with detailed tissue architecture at medium and high resolution, respectively. Automated TIL quantification identifies stromal TILs (sTILs, yellow dots) and intra-tumoral TILs (iTILs, blue dots).

In total, 2418 slides from the same number of patients were available for DIA (Fig. 1). Manual and automated quality control excluded slides with technical artifacts, incomplete data, or insufficient tumor tissue. Stromal and intraepithelial TILs were quantified using the Visiopharm TILs APP (Visiopharm A/S, Hørsholm, Denmark, version 2025.01.0.17336), which segments tumor, stroma, non-invasive epithelium, and necrosis, and detects lymphocytes within tumor-associated stromal and intraepithelial regions (Fig. 5)^23^. Since the APP was originally developed on a TNBC cohort from another department^23^, fine-tuning was required. For this purpose, 41 slides were selected from a corresponding number of patients and annotated by an expert pathologist using the APP’s Author module. These cases were excluded from statistical analyses.

The tumor detection module was fine-tuned with DeepLab V3+ as the underlying network for an additional 75,000 iterations using a learning rate of 1.0 e-07. During the fine-tuning process, several data augmentation steps were applied as described previously^23^. The cellular detection module was used as an off-the-shelf product, and the only refinement made was the incorporation of intraepithelial TILs detection. Since many tumors are well-differentiated and tumor cell nuclei are difficult to distinguish from TILs, a higher threshold was used when classifying cells as a TIL (probability of ≥0.5 in tumor compared to 0.05 in stromal regions was considered positive). This threshold was chosen based on feedback from expert pathologists’ assessment of model performance when testing different cut-off values.

Stromal TIL density (sTIL) was defined as the number of TILs per 0.01 mm² of tumor-associated stroma, while intraepithelial TIL density (iTIL) was defined as the number of TILs per 0.01 mm² of invasive tumor. Algorithm-fail cases (values outside the 99.5th percentile) were manually reviewed, and ten confirmed miscounts were excluded.

### Manual TIL-scoring (*n* = 252)

Additionally, an expert pathologist evaluated a subset of randomly selected cases (*n* = 252) using a continuous scoring method in accordance with the recommendations of the TILs WG^20^. Scoring was conducted prior to and independently of the output from Visiopharm’s APP and later compared to the DIA-derived stromal TIL density to evaluate the level of agreement between the pathologist and DIA. Agreement between pathologist-assessed sTIL percentage and AI-derived sTIL density was quantified using the intraclass correlation coefficient (ICC, two-way mixed-effects model, absolute agreement) and Spearman’s rank correlation. Prior to ICC computation, each variable was independently normalized to a common [0,1] scale using min-max transformation, i.e., (x − min(x))/(max(x) − min(x)), in order to address the unit mismatch between methods (percentage vs. cells/mm²). Neither metric can be interpreted as evidence of absolute equivalence, since the Spearman rank correlation reflects concordance in rank ordering, and the ICC reflects agreement in the rescaled [0,1] domain, and its estimate is sensitive to the observed range of each variable.

### Endpoints

This study focused on distant recurrence (DR) as the primary outcome, defined as the time from initial BC surgery to the occurrence of distant metastasis or death due to BC. Secondary outcomes included time to any recurrence (TR) and overall survival (OS). TR was defined as the duration from surgery to the first event of loco-regional or distant recurrence, or death from BC. OS captured the time from surgery until death from any cause. Survival data were obtained through linkage with the Danish Central Population Registry, with follow-up complete through February 1, 2024. In the analysis of DR and TR, contralateral BC, other primary malignancies, and non–BC-related deaths were treated as competing events^44^. The median follow-up time was 9.2 (IQR: 7.1;10.1) years for DR and TR and 21.9 (IQR: 21.0;22.9) years for OS, as estimated by the inverse Kaplan–Meier method. At 10 years, 237 patients had DR, and 320 had TR. During the study follow-up period, 1370 patients died.

### Statistical analysis

Associations between clinicopathological characteristics and TILs were analyzed using the Chi-squared test for categorical and the Mann–Whitney test for continuous variables. We divided data into sTIL and iTIL high and low categories by splitting at the median. The use of the median as the dichotomization threshold was prespecified, but while the resulting numeric value is inherently data-driven, this approach avoids outcome-driven cutpoint optimization and is distinct from post hoc threshold selection. Categorical analyses are nonetheless secondary to the continuous variable analyses. Analyses for DR, TR, and OS included both univariable and multivariable approaches. To account for competing events in DR and TR, cumulative incidence functions were estimated, and sHRs were derived using Fine–Gray’s model. For OS, survival probabilities were estimated using the Kaplan–Meier method, and HRs were derived from the Cox proportional hazards model. Covariates incorporated into the multivariable models included age (cont.), vascular invasion status (categorical, positive or negative), number of positive lymph nodes (cont. with values 0, 1, 2, or 3), tumor size (cont., log-transformed), ER expression level (cont.), molecular subtype (categorical: Luminal A or B), histological subtype (categorical: ductal, lobular or other), and Nottingham histological grade (cont: 1, 2, or 3). In cases where ER status was reported qualitatively as “Positive” (*n* = 32), and quantitative data were unavailable, a value of 100% was imputed to reflect a high likelihood of positivity and allow inclusion in the models. Covariates were selected a priori based on established prognostic relevance in breast cancer and prior literature, and no data-driven selection algorithm was employed. TIL-associated variables (sTIL and iTIL density) were modeled separately, both as continuous variables (per increase of 1 cell/0.01 mm²) and as dichotomized variables (high vs. low). Continuous predictors were first inspected for nonlinearity using Martingale-residual plots; based on these, tumor size was log-transformed. Proportional hazards (PH) assumptions were assessed by Schoenfeld-residual plots and the *cox.zph* function from the R *survival* package^48^. In the Fine–Gray competing-risk regressions (DR and TR), Nottingham histological grade, ER status, and lymph node status violated the PH assumption and were modeled as piecewise effects (<5 years vs. ≥5 years). In the Cox models for OS, continuous TIL densities violated the assumption and were modeled with a split at 5 years, while age, molecular subtype, and lymphovascular invasion were similarly modelled with a split at 10 years.

To assess whether TILs were associated with outcome in models with a time-dependent factor, we applied a Wald test with 2 degrees of freedom based on the Chi-squared statistic.

Test of heterogeneity for each of the TILs variables according to subtype, nodal status (negative vs positive), and grade, respectively, was applied in separate models using the Wald test, and specifically for continuous TILs in models of OS, by joint Wald test with 2 degrees of freedom (i.e., HR <5, lymph node positive = HR <5, LN-negative and HR ≥5, LN-positive = HR ≥5, LN-negative). Interaction analyses with nodal status and molecular subtype were prespecified in the study protocol. An additional exploratory interaction analysis with histological grade was performed, given its established relationship with immune infiltration in breast cancer^2^. Ki-67 was not systematically available in this cohort and could not be evaluated.

The level of significance was set to 5% and all *p* values reported are two-sided.

### Ethics approval and consent to participate

This study was conducted in accordance with the principles of the Declaration of Helsinki. The Danish Health Research Ethics Committee (reference approval number H-20076778, 09-04-2021, amendment 112835, 03-10-2024) and Region Zealand Research Register (Forskningsfortegnelse, reference approval number REG-088-2022) approved the study. The Danish Registry for Use of Tissue was checked to ensure that no patients included in this study were registered there. The requirement for informed consent was waived by the Danish Health Research Ethics Committee owing to the retrospective design of the study and the use of anonymized archival material.

## Supplementary information

SupplementaryMaterials_R2.

## Data Availability

The datasets generated and analysed during the current study are not publicly available due to institutional policies and Danish Health Act restrictions on the public release of patient-level data. De-identified data may be made available to qualified researchers upon reasonable request, subject to approval by the Danish Breast Cancer Group (DBCG), which is the data controller, and the relevant Danish authorities, and contingent on the establishment of a data-processing/data-transfer agreement in accordance with Danish and European Union law. Requests should be directed to the corresponding author or DBCG.

## Code availability

TIL quantification was performed using the commercial Visiopharm TILs APP (Visiopharm A/S, Hørsholm, Denmark, version 2025.01.0.17336). Statistical analyses were conducted in R using standard, publicly available packages. The custom R analysis scripts are available from the corresponding author on reasonable request.
