# Supplementary material for: AI-assisted image analysis of tumor-infiltrating lymphocytes as a prognostic marker in chemotherapy-naïve luminal breast cancer
Source: NPJ Breast Cancer. 2026 Jul 1;12:123. doi: 10.1038/s41523-026-00999-w (PMC13615976; doi:10.1038/s41523-026-00999-w)
Supplement: Supplementary file 1 [file 41523_2026_999_MOESM1_ESM.pdf]

Title:

AI-assisted image analysis of tumor-infiltrating lymphocytes as a prognostic marker in chemotherapy-naïve luminal breast cancer

Journal: *npj Breast Cancer*

Author list:

Dusan Rasic (1, 2)*#, Sandra Sinius Pouplier (1, 2)*, Maj-Britt Jensen (3), Bent Ejlertsen (2, 3, 4), Roberto Salgado (5, 6), Pekka Ruusuvuori (7), Johan Hartman (8, 9), Mattias Rantalainen (10), Anne-Vibeke Lænkholm (2, 11)

1) Department of Surgical Pathology, Zealand University Hospital, Denmark
2) Department of Clinical Medicine, Faculty of Health and Medical Sciences, University of Copenhagen, Denmark
3) Danish Breast Cancer Group, Department of Oncology, Rigshospitalet, Copenhagen University Hospital, Copenhagen, Denmark
4) Department of Oncology, Centre for Cancer and Organ Disease, Rigshospitalet, Copenhagen University Hospital, Denmark
5) Department of Pathology, ZAS Hospitals, Antwerp, Belgium
6) Division of Research, Peter MacCallum Cancer Centre, Melbourne, Australia
7) Institute of Biomedicine, University of Turku, Kiinamyllynkatu 10, 20520 Turku, Finland
8) Department of Oncology and Pathology, Karolinska Institute, Stockholm, Sweden
9) Department of Clinical Pathology and Cancer Diagnostics, Karolinska University Hospital, Stockholm, Sweden
10) Department of Medical Epidemiology and Biostatistics, Karolinska Institute, Stockholm, Sweden
11) Department of Pathology, Herlev and Gentofte University Hospital, Herlev, Denmark

***Dusan Rasic and Sandra Sinius Pouplier have contributed equally and share first co-authorship**

[#]Corresponding author:
Dusan Rasic, MD
Department of Surgical Pathology, Zealand University Hospital, Denmark
Orcid-ID: 0000-0003-4610-5265
dura@regionsjaelland.dk

Supplementary Figure 1. Scatter plot of DIA sTIL density and pathologist assessed sTIL%

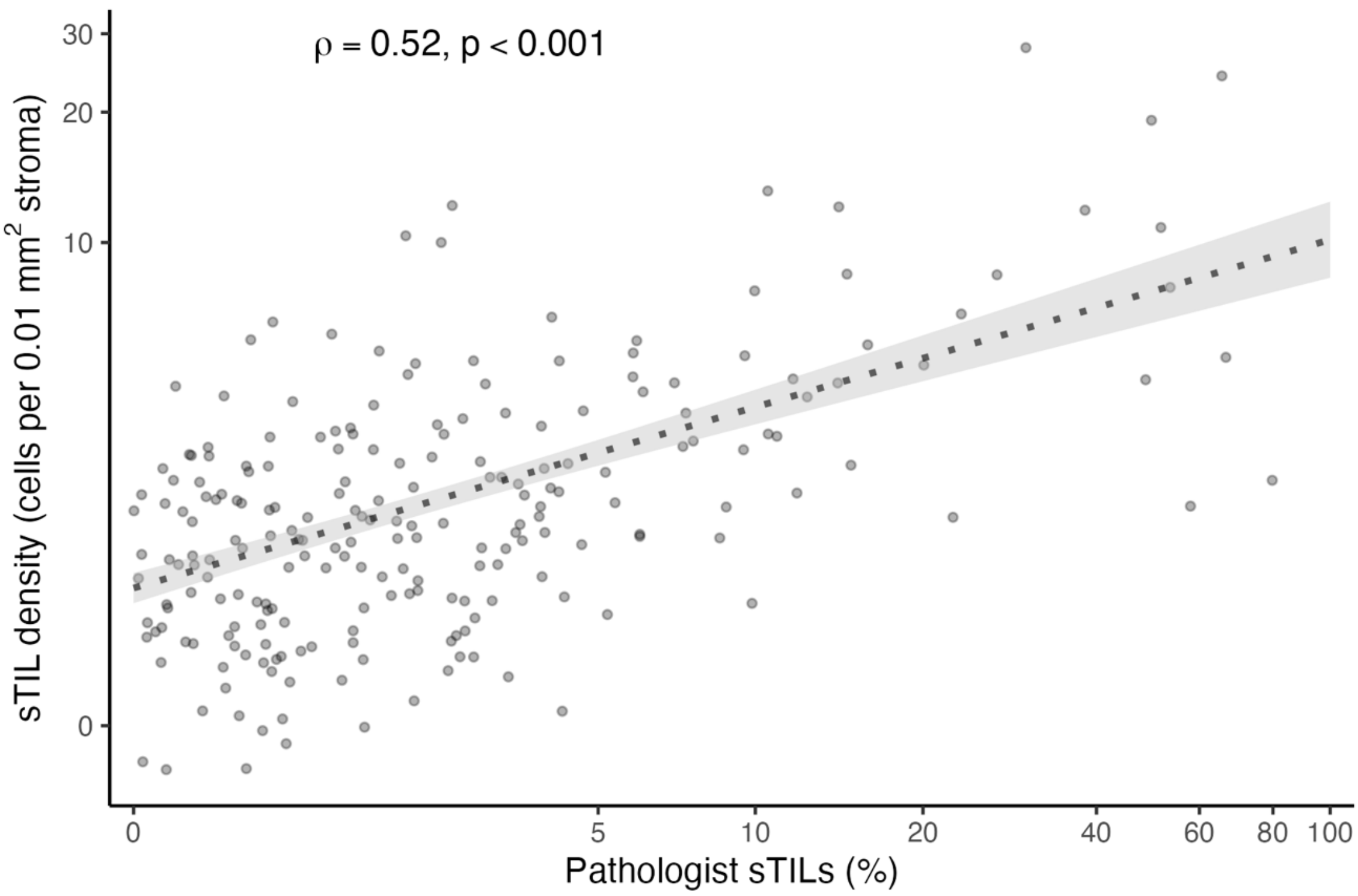

Supplementary Figure 1. Scatter plot of pathologist-assessed stromal TIL (%) versus digital image analysis (DIA)–derived sTIL density (cells per 0.01 mm² stroma). Each point represents one case. The dotted line shows the linear regression fit. The shaded area indicates the 95% confidence interval. Both axes are displayed on a log1p scale.

Supplementary Figure 2. Distribution of sTIL scores by assessment method

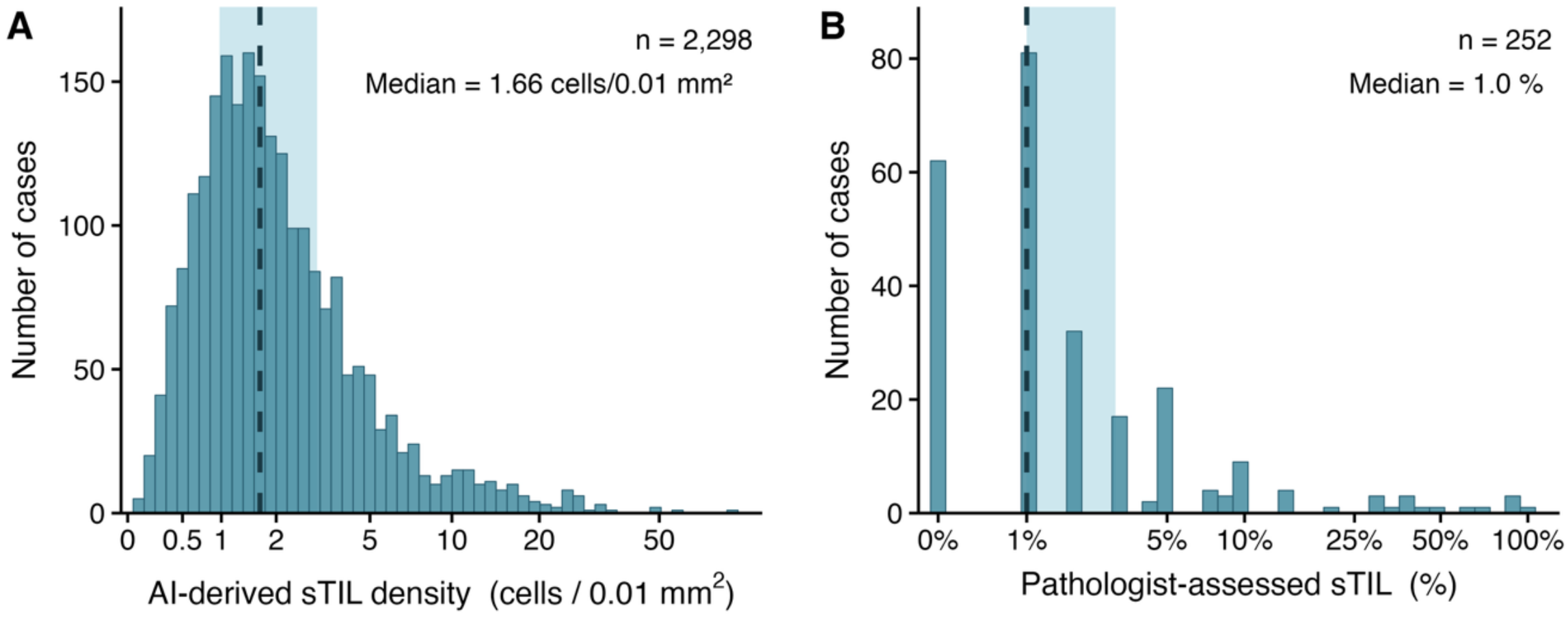

Supplementary Figure 2. Distribution of stromal TIL scores by assessment method. (A) Histogram of AI-derived sTIL density (cells/mm² of tumour-associated stroma) for the full analysis cohort (n = 2,298). (B) Histogram of pathologist-assessed sTIL percentage (%) for the manually scored subset (n = 252). Both panels are displayed on a log1p-transformed x-axis to accommodate the right-skewed distribution. The vertical dashed line indicates the median; the shaded band indicates the interquartile range.

Supplementary Table 1. Clinicopathological characteristics of luminal A vs. luminal B BC in our cohort

Variable	Level	Luminal A (n=1,390)	Luminal B (n= 908)	p-value
Age (years)	50-59	537 (64.3)	298 (35.7)	<0.01
	60-69	595 (61.0)	381 (39.0)	
	≥70	258 (53.0)	229 (47.0)	
Vascular invasion	No	1,269 (61.9)	780 (38.1)	<0.01
	Yes	121 (48.6)	128 (51.4)	
Positive lymph nodes	0	572 (55.3)	462 (44.7)	<0.01
	1	472 (67.0)	232 (33.0)	
	2	231 (64.0)	130 (36.0)	
	3	115 (57.8)	84 (42.2)	
Tumor size (mm)	≤10	169 (79.3)	44 (20.7)	<0.01
	11-20	638 (65.0)	343 (35.0)	
	21-30	407 (52.4)	370 (47.6)	
	>30	176 (53.8)	151 (46.2)	
NHG	1	507 (78.1)	142 (21.9)	<0.01
	2	834 (59.6)	566 (40.4)	
	3	49 (19.7)	200 (80.3)	
Estrogen receptor	10-59	144 (69.6)	63 (30.4)	0.02
	60-89	296 (63.4)	171 (36.6)	
	90-99	348 (57.5)	257 (42.5)	
	100	585 (58.9)	408 (41.1)	
	Positive*	17 (65.4)	9 (34.6)	
Histological subtype	Ductal	1,110 (58.2)	798 (41.8)	<0.01
	Lobular	241 (76.5)	74 (23.5)	
	Other	39 (52.0)	36 (48.0)	
sTILs (cont)*	median [iqr]	1.57 [0.92, 2.69]	1.87 [1.06, 3.69]	<0.01
sTILs (cat)	Low	744 (64.8)	405 (35.2)	<0.01
	High	646 (56.2)	503 (43.8)	
iTILs (cont)*	median [iqr]	0.23 [0.11, 0.50]	0.19 [0.09, 0.48]	0.05
iTILs (cat)	Low	663 (57.7)	486 (42.3)	<0.01
	High	727 (63.3)	422 (36.7)	

Values in the parentheses are given as row percentages for categorical variables and as interquartile ranges for continuous variables. *TIL densities, stromal and intraepithelial TIL density expressed as cells per 0.01 mm^2 of tumor-associated stroma and tumor, respectively (digital image analysis).
*Value on continuous scale unknown.
Abbreviations: sTILs, stromal tumor-infiltrating lymphocytes. iTILs, intraepithelial tumor-infiltrating lymphocytes. NHG, Nottingham histological grade.

Supplementary Table 2. Associations between distant recurrence, any recurrence, and overall survival with sTILs and iTILs (continuous): multivariable analyses with interaction by grade as continuous variable. Recurrence is analysed with Fine-Gray subdistributional hazard model and results are reported with subdistribution hazard ratios (sHR). Overall survival is analysed with a Cox proportional hazards model – results are hazard ratios (HR).

**Subgroup analysis – histological grade**								
		**DR**		**TR**		**OS**		
**Variable**	**Grade**	**sHR** (95% CI)	**p** heterogeneity	**sHR** (95% CI)	**p** heterogeneity	**HR** (95% CI)		**p** heterogeneity
sTILs (cont.)	1	0.96 [0.87;1.06]	0.87	0.94 [0.86;1.04]	0.99	<5y	0.92 [0.86;0.99]	0.30
						≥5y	1.00 [0.97;1.03]	
	2	0.95 [0.91;1.00]		0.95 [0.91;0.99]		<5y	0.91 [0.85;0.97]	
						≥5y	0.99 [0.97;1.00]	
	3	0.95 [0.88;1.02]		0.95 [0.89;1.02]		<5y	0.90 [0.84;0.96]	
						≥5y	0.98 [0.95;1.00]	
iTILs (cont.)	1	0.88 [0.61;1.28]	0.69	0.81 [0.55;1.19]	0.98	<5y	0.88 [0.70;1.10]	0.49
						≥5y	1.05 [0.95;1.16]	
	2	0.83 [0.66;1.04]		0.80 [0.64;1.01]		<5y	0.85 [0.69;1.04]	
						≥5y	1.02 [0.95;1.08]	
	3	0.78 [0.52;1.16]		0.80 [0.54;1.19]		<5y	0.82 [0.65;1.03]	
						≥5y	0.98 [0.85;1.13]	

$p_{\text{hetereogeneity}}$ indicates Wald test with 1 degree of freedom for all endpoints whether the prognostic effect of sTILs/iTILs changes linearly across histological grade (Grade 1, 2, 3); p<0.05 indicates evidence that the effect differs by grade. For OS, hazard ratio estimates are presented separately for the early (<5 years) and late (≥5 years) follow-up periods, consistent with the time-varying TIL effects established in the main analysis; the interaction p-value reflects a single test of the TIL × grade association across both time periods (i.e. HR[Grade 1] = HR[Grade 2] = HR[Grade 3]).

Abbreviations: DR, distant recurrence. TR, any recurrence. OS, overall survival. sHR, subdistribution hazard ratio. HR, hazard ratio.
